# Candidaemia and a risk predictive model for overall mortality: a prospective multicentre study

**DOI:** 10.1186/s12879-019-4065-5

**Published:** 2019-05-21

**Authors:** C. Keighley, S. C-A. Chen, D. Marriott, A. Pope, B. Chapman, K. Kennedy, N. Bak, N. Underwood, H. L. Wilson, K. McDonald, J. Darvall, C. Halliday, S. Kidd, Q. Nguyen, K. Hajkowicz, T. C. Sorrell, S. Van Hal, M. A. Slavin

**Affiliations:** 1 Centre for Infectious Diseases and Microbiology Laboratory Services, ICPMR, New South Wales Health Pathology, Westmead Hospital, Darcy Rd, 3rd Level, ICPMR Building, Westmead, Sydney, New South Wales 2145 Australia; 2 Marie Bashir Institute for Infectious Diseases and Biosecurity, The University of Sydney, Sydney, NSW Australia; 3 Department of Infectious Diseases, Westmead Hospital, Westmead, Sydney, NSW Australia; 4 Department of Microbiology and Infectious Diseases, St. Vincent’s Hospital, Sydney, NSW Australia; 5 Eastern Health Clinical School, Monash University, Melbourne, Victoria Australia; 6 School of Mathematics and Statistics, University of NSW, Sydney, NSW Australia; 7 Westmead Institute for Medical Research, Westmead, NSW Australia; 8 Department of Infectious Diseases and Microbiology, Canberra Hospital, Australian National University Medical School, Canberra, ACT Australia; 9 Department of Infectious Diseases, Royal Adelaide Hospital, Adelaide, SA Australia; 10 Infection Management Services, Princess Alexandra Hospital, Brisbane, QLD Australia; 11 Department of Intensive Care, Royal Melbourne Hospital, Melbourne, VIC Australia; 12 National Mycology Reference Centre, SA Pathology, Adelaide, SA Australia; 13 National Centre for Clinical Excellence on Emerging Drugs of Concern (NCCRED), National Drug and Alcohol Research Centre (NDARC), University of New South Wales, Sydney, Australia; 14 Department of Infectious Diseases, Royal Brisbane and Women’s Hospital, School of Medicine, University of Queensland, Brisbane, QLD Australia; 15 Department of Infectious Diseases and Microbiology, New South Wales Health Pathology, Royal Prince Alfred Hospital, Sydney, NSW Australia; 16 Department of Infectious Diseases, Peter MacCallum Cancer Centre, National Centre for Infections in Cancer, Melbourne, VIC Australia

**Keywords:** Candidaemia, Candida blood stream infection, Mortality, Risk stratification score, Invasive fungal infection

## Abstract

**Background:**

Candidaemia is associated with high mortality. Variables associated with mortality have been published previously, but not developed into a risk predictive model for mortality. We sought to describe the current epidemiology of candidaemia in Australia, analyse predictors of 30-day all-cause mortality, and develop and validate a mortality risk predictive model.

**Methods:**

Adults with candidaemia were studied prospectively over 12 months at eight institutions. Clinical and laboratory variables at time of blood culture-positivity were subject to multivariate analysis for association with 30-day all-cause mortality. A predictive score for mortality was examined by area under receiver operator characteristic curves and a historical data set was used for validation.

**Results:**

The median age of 133 patients with candidaemia was 62 years; 76 (57%) were male and 57 (43%) were female. Co-morbidities included underlying haematologic malignancy (*n* = 20; 15%), and solid organ malignancy in (*n* = 25; 19%); 55 (41%) were in an intensive care unit (ICU). Non-*albicans Candida* spp*.* accounted for 61% of cases (81/133). All-cause 30-day mortality was 31%. A gastrointestinal or unknown source was associated with higher overall mortality than an intravascular or urologic source (*p* < 0.01). A risk predictive score based on age > 65 years, ICU admission, chronic organ dysfunction, preceding surgery within 30 days, haematological malignancy, source of candidaemia and antibiotic therapy for ≥10 days stratified patients into < 20% or ≥ 20% predicted mortality. The model retained accuracy when validated against a historical dataset (*n* = 741).

**Conclusions:**

Mortality in patients with candidaemia remains high. A simple mortality risk predictive score stratifying patients with candidaemia into < 20% and ≥ 20% 30-day mortality is presented. This model uses information available at time of candidaemia diagnosis is easy to incorporate into decision support systems. Further validation of this model is warranted.

**Electronic supplementary material:**

The online version of this article (10.1186/s12879-019-4065-5) contains supplementary material, which is available to authorized users.

## Background

Candidaemia (or bloodstream infection with *Candida* spp.) continues to increase and ranks amongst the top 5 hospital-acquired infections in many countries [1–5]. The shift in aetiology of candidaemia towards non-*albicans Candida* spp. and in particular, the disproportionate increase of *Candida glabrata* complex infections is of concern [2, 4]. Resistance to azole and echinocandin antifungal drugs, though increasing in certain regions, is uncommon in Australia [1]. Mortality from candidaemia remains high (up to 40%) with prolonged hospital stay and excess costs [1–3, 5–7].

Better understanding of the variables that influence mortality is essential to improving outcomes in patients with candidaemia. A number of studies have found that mortality increases with age (e.g. > 65 years), admission to an intensive care unit (ICU), use of total parenteral nutrition (TPN) or broad-spectrum antibiotics, organ dysfunction and a gastrointestinal source of candidaemia [6, 8–11]. In addition, delays in source control and initiation of appropriate antifungal therapy adversely affect outcomes [12, 13].

Despite a number of models using risk stratification to predict the likelihood of developing candidaemia [11, 14], to our knowledge, there is no similar stratification model to predict mortality. Stratification of the risk of mortality may help delineate patients in whom an aggressive approach to source control is needed, or, given that therapy with echinocandins requires intravenous therapy and hospitalisation, may guide an earlier transition to oral therapy [15, 16].

We conducted a contemporary multicentre, prospective study of the epidemiology and complications of candidaemia in Australia and assessed factors influencing mortality. Based on these data we propose a simple risk prediction model for overall mortality using clinical and laboratory variables known at the time of notification of a positive blood culture.

## Methods

### Study design and data collection

The study was a prospective observational study at eight tertiary referral hospitals carried out between March 2014 and February 2016. Patients were identified through active laboratory-based surveillance at each centre over a total of 12 months from time of commencement. Human research ethics approval was obtained with study oversight through the Western Sydney Local Health District HREC (HREC Ref: AU RED LNR14/WMEAD/112).

All adults ≥18 years, with at least one blood culture positive for *Candida* spp., were enrolled. Episodes of recurrent candidaemia (occurring more than 30 days after the initial episode) within the study period were excluded. Cardiac echocardiography and an ophthalmology assessment were performed as directed by the treating clinician. Data collected on standardised case report forms included: patient demographics, healthcare setting, patient co-morbidities (e.g. malignancy, diabetes mellitus; Additional file 1), presence of a predisposing factor in the preceding 30 days (e.g. surgery and type thereof, central vascular access device [CVAD]), likely source of candidaemia (see Definitions below and in Additional file 1), complications, results of laboratory studies, and treatment and clinical outcomes at 30 days. All data were collected at baseline with progress including treatment and outcome at day 7 and 30 after the date of the initial positive blood culture or at death if this occurred earlier. Mortality was defined as due to candidaemia unless another identifiable cause was ascribed as per the treating physician. Periodic audits ensured complete case capture.

### Definitions

A case (or episode) was defined as isolation of one or more *Candida* spp. from blood during the study period. The date of candidaemia was the date of first positive blood culture. Inpatient healthcare associated (IHCA) candidaemia were defined as episodes that occurred >/= 48 h after hospital admission and which were not clinically manifest on admission. Among cases classed as outpatient-acquired candidaemia, episodes associated with recent healthcare contact events e.g. surgical procedures, were classed as outpatient healthcare associated episodes (OHCA) whilst cases with no healthcare –related risk factors were community–acquired (CA) [6]. The source of candidaemia was determined by the attending physician as i) intravascular (same *Candida* species isolated from the tip of an intravascular device as that from blood culture, or physician-ascribed where indwelling intravascular device was the only likely source, or candidaemia in the context of documented intravenous drug use as the only likely source); ii) gastrointestinal (same *Candida* species identified from specimens originating from a gastrointestinal or intra-abdominal source as that from blood culture or physician-ascribed where documented interventional procedure e.g. gastrointestinal surgery, biliary tract manipulation and no other likely source); iii) urologic (same *Candida* species isolated from urine as that from blood culture following antecedent urologic instrumentation or surgery that clearly preceded candidaemia and no other likely source); or iv) unknown (not attributable to an intravascular, gastrointestinal or urologic source) (see table included in Additional file 1). Sepsis at the time of blood culture collection was defined according to the Australian national sepsis guidelines [17]. Endocarditis was classified by modified Duke criteria [18]. Neutropenia was defined as a neutrophil count of < 1.0 X 10^9^ cells/L.

### Microbiological methods

Blood was cultured in BACTEC (Becton Dickinson, Sparks, MD, USA) or BacT/Alert 3D (bioMérieux, Marcy l’Etoile, France) blood culture automated systems. *Candida* organisms were identified to species level by matrix-assisted laser desorption/ionization- time of flight mass spectrosocopy (MALDI-TOF MS) (Biotyper database v 3.1; Bruker Daltoniks, Germany) or by Vitek 2 YST, 20C AUX or ID 32C identification systems (bioMérieux, Marcy-L’Étoile, France). All isolates were forwarded to a mycology reference laboratory (Westmead Hospital, Sydney, New South Wales or SA Pathology, Adelaide, South Australia) for species confirmation by internal transcribed spacer (ITS) sequencing [19] and for antifungal susceptibility testing [20, 21]. Where species identification was discordant the reference laboratory determination was used.

### Statistical analysis

Data were analyzed with R version 2.15.13 (R Core Team, Vienna, Austria). For univariate analysis, continuous variables were compared with the student *t* test, and categorical variables compared with the two-tailed Chi-squared test with Yates continuity correction as indicated. A *p* value < 0.05 was considered significant. Odds ratios and 95% confidence intervals (CI) were calculated. Kaplan-Meier and log-rank test analyses were used to test for an association between the source of candidaemia and mortality.

In order to produce a model which could be used to guide further treatment, input variables were restricted to those known at the time of positive blood culture. Variables with *p* < 0.15 for death at 30 days on univariate analysis were included in the multivariate logistic regression model. For simplicity of use in hospital wards, all variables, including source of candidaemia, were converted to binary variables. In order to reduce the effect of possible over-fitting (otherwise known as optimism bias) multiple random subsets consisting of 80% of the sample were used to construct prediction models. These models were refined by a stepwise elimination procedure and then tested on the remaining 20% of the sample to evaluate performance. Variables which were important in a large proportion of these random test samples were retained in the final model. The final model was then tested against a historical national dataset of 741 prospectively recorded cases (2001–2004) [6].

## Results

### Patient demographics

There were 138 incident cases of candidaemia (96%) of which 133 were evaluable (in five patients, data to 30 days were incomplete). The median patient age was 62 years (interquartile range, IQR 51–73); 76 (57%) were male and 57 (43%) were female (see Table 1). The proportion of patients with IHCA, OHCA and CA candidaemia is shown in Table 1. For 55 patients (41%) in ICU at the time of diagnosis, the median APACHE (Acute physiology and chronic health evaluation) II score for ICU patients was 19 (IQR 16–27) and the median length of ICU stay was 7 (IQR 2–14) days.

**Table 1:** Characteristics of patients with candidaemia and univariate analysis for all cause 30-day mortality

	No. patients (total = 133)	30-day mortality	Odds ratio, 95% confidence interval	*P* value
Age > 65 years	57 (43)	24 (42)	2.7 (1.3–5.8)	0.01
Male gender	76 (57)	24 (32)	1.1 (0.5–2.3)	0.8
Female gender	57 (43)	17 (30)	0.9 (0.4–1.9)	0.8
Setting of candidaemia				
IHCA	113 (85)	35 (31)	1 .0 (0.4–3.0)	0.9
OHCA	13 (10)	3 (23)	0.6 (0.2–2.5)	0.6
CA	7 (5)	3 (43)	0.6 (0.2–2.5)	0.5
Admitting service				
Medical	77 (58)	30 (39)	2.6 (1.2–5.8)	0.02
Surgical	56 (42)	11 (20)	0.4 (0.2–0.8)	0.02
ICU admission	55 (41)	22 (40)	2.1 (1.0–4.4)	0.06
Co-morbidities				
Haematologic malignancy	20 (15)	10 (50)	2.6 (1.0–7.0)	0.06
Allogeneic stem cell transplantation	2 (1)	1 (50)	2.3 (0.1–37.3)	0.6
Solid organ malignancy	25 (19)	7 (28)	0.8 (0.3–2.2)	0.7
Solid organ transplantation	5 (4)	1 (20)	0.5 (0.1–5.0)	0.6
Diabetes mellitus	33 (25)	10 (30)	1.0 (0.4–2.3)	0.9
Chronic organ dysfunction	50 (38)	29 (58)	0.4(0.2–0.9)	0.03
Renal disease	18 (14)	8 (44)	2.0 (0.7–5.5)	0.2
Liver disease	15 (11)	5 (33)	1.1 (0.4–3.6)	0.8
Cardiovascular disease	26 (20)	11 (42)	1.8 (0.8–4.6)	0.2
Respiratory disease	11 (8)	5 (45)	2.0 (0.6–6.9)	0.3
Predisposing factors				
Surgery	69 (52)	16 (23)	0.5 (0.2–1.0)	0.05
Gastrointestinal surgery	35 (26)	11 (31)	1.0 (0.4–2.4)	0.9
Urinary catheter	80 (60)	15 (19)	1.6 (0.7–3.6)	0.2
CVAD	98 (74)	30 (31)	1.0 (0.4–2.2)	0.9
Hyperalimentation	27 (20)	5 (19)	0.4 (0.2–1.3)	0.1
Corticosteroids or other immunosuppressant	36 (27)	13 (36)	1.4 (0.6–3.1)	0.4
Intravenous drug use	13 (10)	1 (8)	0.2 (0.02–1.3)	0.1
Use of antibiotic agents ≥10 days	39 (29)	16 (41)	1.9 (0.9–4.2)	0.1
Prior antifungal use^a^	19 (14)	7 (37)	1.4 (0.5–3.8)	0.5
Sepsis syndrome	97 (73)	29 (30)	0.9 (0.4–1.9)	0.5
Source of candidaemia				
Intravascular	42 (32)	9 (21)	0.5 (0.2–1.2)	0.1
Gastrointestinal	46 (35)	19 (41)	2.1 (1.0–4.4)	0.06
Urologic	27 (20)	2 (7)	0.1 (0.03–0.6)	0.009
Unknown	18 (14)	11 (61)	4.4 (1.6–12.5)	0.005
Candida species				
*Candida albicans*	52 (39)	17 (33)	1.2 (0.5–2.4)	0.7
*Candida glabrata* complex	43 (32)	15 (35)	1.4 (0.5–3.8	0.5
*Candida parapsilosis* complex	13 (10)	1 (8)	0.2 (0.02–1.3)	0.09
Other *Candida* species ^b^	25 (19)	8 (32)	1.1 (0.4–2.7)	0.9

Counts are shown as n (%) unless otherwise stated

*CA* community acquired, *CVAD* Central venous access device, *IHCA* inpatient healthcare associated, *ICU* intensive care unit, *OHCA* outpatient healthcare associated

^a^ Antifungals used prior to diagnosis of candidaemia included fluconazole in 15 patients, voriconazole in 1, anidulafungin in 1, caspofungin as prophylaxis in 2 and voriconazole for treatment of possible pulmonary aspergillosis in 1

^b^ *C. tropicalis* (*n* = 10), *C, krusei* (*n* = 5), *C. robusta* (n = 1), *C. dubliniensis* (n = 1), *C. lipolytica* (n = 1), *C. lusitaniae* (n = 1), more than 1 *Candida spp.* (*n* = 6); these were *C. albicans* and *C. glabrata* sensu stricto (n = 2), *C. albicans* and *C. bracariensis* (*n* = 1), *C. albicans* and *C. nivariensis* (n = 1), *C. albicans* and *C. parapsilosis* sensu stricto (n-1), and *C. albicans,C. parapsilosis* sensu stricto and *C. tropicalis* (n = 1)

### Patient characteristics

Summary statistics for demographic and risk factors in the cohort are presented in Table 1, as are the major co-morbidities and predisposing factors for candidaemia in the patient cohort.

There were 53 patients with two or more co-morbidities (40%) and 35 patients (25%) with none. The most common co-morbidity was diabetes mellitus (33 patients, 25%) followed by cardiovascular disease (26 patients, 20%) and solid organ malignancy (25 patients, 19%) (Table 1). Of patients with solid organ malignancy, 7 were in ICU at the time of candidaemia diagnosis. Three had received cytotoxic chemotherapy and 18 had undergone surgery within the past 30 days. Of 20 patients with a haematological malignancy, 12 were neutropenic. Two patients had received an allogeneic stem cell transplant (SCT); one had received fluconazole for 21 days at the time of *C. krusei* candidaemia and the other had received liposomal amphotericin for more than 30 days at the time of *C. albicans* candidaemia.

Common predisposing factors included a central venous access device (CVAD; *n* = 98, 74%) and a urinary catheter (*n* = 80, 60%). CVADs had been in situ for a median of 8 (IQR 3–17) days with 13 (13%) in place for > 30 days. Urinary catheters had been in situ for a median of 6 (IQR 2–15) days with 10 (13%) in place for > 30 days. Hyperalimentation with TPN was administered to 27 patients (20%), for a median 13 (IQR 5–20) days. There were 110 patients with at least 2 predisposing factors (83%) and seven patients (5%) with none. The latter included two patients with neither a co-morbidity nor a predisposing factor.

Antibacterial drugs were administered to 119 patients (89%) for a median of 5 (IQR 1–11) days prior to diagnosis of candidaemia and 58% received two or more antibiotics (Table 1). There was no association between any particular antibiotic and *Candida* spp. on univariate or multivariate analysis. Twenty patients developed candidaemia (Table 1) whilst receiving a prophylactic antifungal agent (median 7 days [IQR 3–16]). In 16/20 patients, the *Candida* species was susceptible or wild-type to the prior antifungal agent used.

### Microbiology

*Candida albicans* caused 52 (39%) episodes followed by *C. glabrata* complex (43, 32%) and *C. parapsilosis* complex (13, 10%) (Table 1). Non-*albicans Candida* spp. predominated; non-*albicans Candida* spp. accounted for 58% (65/112) of IHCA, 84% (11/13) of OHCA and 63% (5/8) of CA episodes. Isolation of the *C. glabrata* complex was not associated with age > 65 years, or with particular co-morbidities or predisposing factors (data not shown). Prior antifungal use (*n* = 20) did not correlate with *Candida* spp. identified in this context.

*C. glabrata* complex was isolated significantly more frequently from patients with a probable gastrointestinal source than alternative sources (20/46, 43% vs. 23/87, 26%; OR 2.14 (95%CI 1.01–4.54); *p* = 0.05). Conversely, *C. parapsilosis* complex was more common in candidaemia associated with an intravascular source compared with a source classed as “non-intravascular” (9/42, 21% vs. 4/91, 44%; OR 5.93 (95%CI 1.71–20.58; *p* = 0.002). Recovery of *C. albicans* was equally likely regardless of attributable source (16/42, 38% intravascular, 15/46, 33% gastrointestinal, 12/27, 44% renal tract, 9/18, 50% unknown).

### Complications

Complications of candidaemia were documented in 10% (13/133) of episodes. Endocarditis was identified in 5% (6/110) of patients undergoing echocardiography and endophthalmitis in 6% (6/98) of patients undergoing ophthalmological assessment. One patient had hepatosplenic candidiasis. There were no cases of fungal meningitis. Mortality at 30 days for patients with these complications was 5/13 (38%).

### Therapy

Amongst 117 patients who received antifungal therapy at diagnosis of candidaemia, 54 (46%) were commenced on fluconazole and 60 (51%) an echinocandin at doses concordant with published guidelines [15]. Sixteen patients (12%) received only palliative therapy (with no antifungals) or died prior to treatment. Seventy-eight of 133 (59%) episodes were due to isolates which were susceptible to fluconazole and of the patients who received fluconazole as empiric therapy, 46/54 (85%) of isolates were susceptible to fluconazole. All isolates were susceptible to echinocandins. The median duration of antifungal therapy for uncomplicated candidaemia was 15 days (IQR 14–22 days).

The 30-day mortality in patients who received fluconazole (10/54, 19%) vs an echinocandin (14/60, 23%) was similar (data not shown; *p* = 0.5), after adjusting for variables identified in our mortality risk predictive score and *Candida* species. Only two of the patient deaths occurring in the initial fluconazole-treated group was associated with non-susceptibility to fluconazole.

Nineteen patients who received an echinocandin as initial antifungal therapy were stepped down to fluconazole after a median of 4 days (IQR 2–5); 11 (58%) patients had therapy de-escalated after 4 days or less (9 after identification of *Candida* species and 2 after susceptibility results). There was no correlation between shorter time to step-down and outcome.

### Outcome

**Fig. 1:**
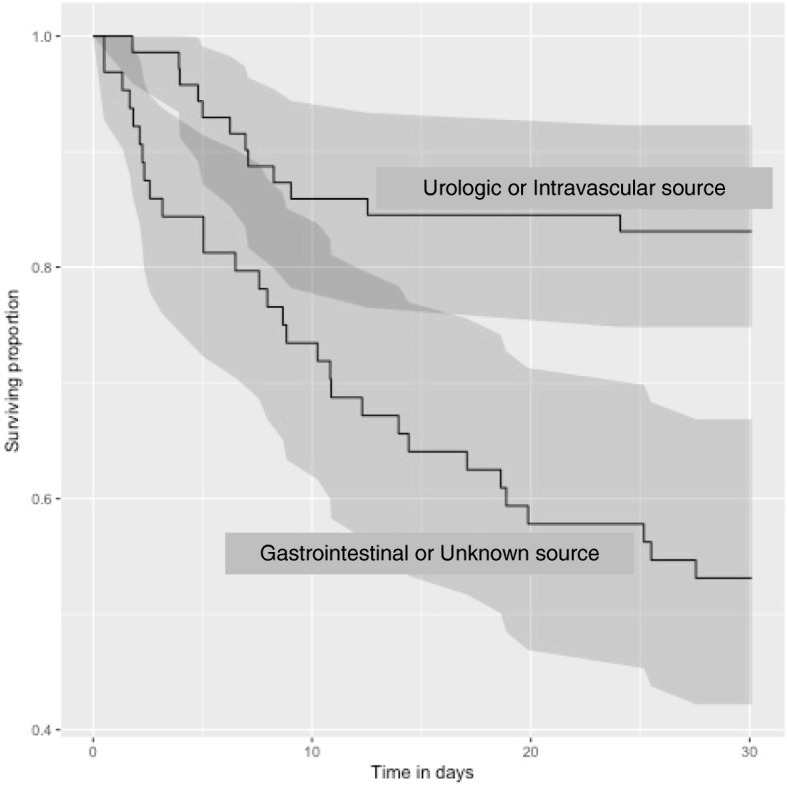
Kaplan-Meier survival curve of all patients with candidaemia stratified by source; genitourinary/intravascular or gastrointestinal/unknown. Source was grouped to produce a binary variable of favourable versus unfavourable outcome. Log-rank test statistic for equality of curves: chi-squared = 6.7 on 1 degrees of freedom, *p* = 0.00967. Shaded areas are pointwise 95% confidence intervals, showing the curves are well-separated

The 7-day overall mortality was 21% (28/133), and 30-day mortality, 31% (41/133) with a candidaemia-attributed mortality of 13% (17/133) as per treating physician. At 30 days, the condition of only 66 patients (50%) was either resolved or improved as judged by their physician. Mortality was lower in patients with an intravascular or urologic source of candidaemia than in patients with a gastrointestinal or unknown source (*p* < 0.01) (Fig. 1). CVADs had been removed within 72 h of the diagnosis of candidaemia in 87% (85/98); time to removal of CVAD was not significantly correlated with mortality. Sepsis was present in 73% (97/133) and was not correlated with mortality (Table 1).

### Mortality risk predictive score

**Fig. 2:**
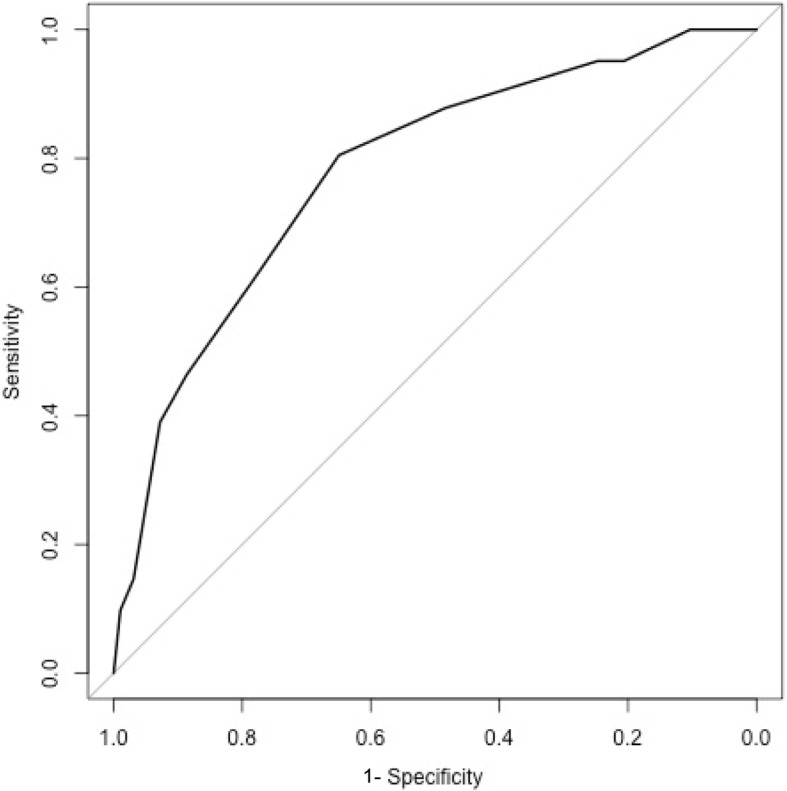
Area under the curve (AUC) = 0.81 for derivation cohort. When applied to *n*=741 historical cohort data (2001-2004), AUC = 0.74

The univariate analysis is summarised in Table 1. By multivariate logistic regression analysis, mortality was associated with age > 65 years, presence in ICU at the time of diagnosis of candidaemia, a haematological malignancy, organ failure within the prior 30 days, absence of recent surgery, receipt of antibiotics for ≥10 days at diagnosis of candidaemia and an abdominal or unknown source of candidaemia (Table 2, Additional file 1). The APACHE II score was not included as a variable in the risk predictive score as it was only available for patients admitted to ICU. For in-sample prediction the AUC was 0.81. The score was out of a maximum of 7.5 points with higher points equating to higher mortality. All coefficients were close to 1 or 1.5, therefore, to produce a tool that could be calculated with minimum difficulty on the ward, the coefficients were adjusted to produce the Score column in Table 2. Using these approximate coefficients in the final logistic regression model gave predictions close to those obtained using the original coefficients (AUC 0.80, see Fig. 2). A score of 0 had a negative predictive value (NPV) of 100%; there were 0 deaths amongst 10 patients in the current dataset.

**Table 2:** Risk prediction model for all cause 30-day mortality

	Coefficient	SE	OR	95% CI of OR	Score
Age > 65 years	1.4	0.5	3.8	1.6–10.0	1.5
Location in ICU at time of diagnosis	0.9	0.5	2.5	1.0–6.4	1
No prior surgery	1.0	0.5	2.6	1.0–6.9	1
Any haematological malignancy	1.0	0.6	2.8	0.85–9.4	1
Chronic organ dysfunction^a^	1.0	0.5	2.7	1.1–6.6	1
Gastrointestinal or unidentified attributable source	0.9	0.4	2.5	1.1–6.1	1
Use of antibiotic agents ≥10 days	1.0	0.5	2.6	1.0–6.7	1

^a^ One or more of renal disease, liver disease, cardiovascular disease or respiratory disease (Additional file 1)

*SE* Standard error of coefficient, *OR* Odds ratio, *CI* Confidence interval

As all coefficients were close to 1 or 1.5, the coefficients were adjusted to produce the Score column

In order to produce a binary prediction for each individual, risk predictive scores were grouped by assigning individuals with risk predictive scores in the range 0 to 2 inclusive to the “lower-risk” category. According to the model, mortality in this group was less than 20% at 30 days. The threshold of 2 was chosen because this was a natural break in the data: patients with risk predictive scores greater than this had at least 33% mortality at 30 days, according to the model. There was little change in the Positive Predictive Value (PPV) over the risk predictive score range 1 to 2 (Table 3).

**Table 3:** Performance of the mortality prediction model for individual score values

Score	6.5	5.5	5	4.5	4	3.5	3	2.5	2	1.5	1	0
Cumulative fraction with value ≥ to Score	0.7	5.2	8.2	17.9	23.1	36.6	53.0	63.4	82.1	85.1	92.5	100.0
PPV	100	86	73	71	65	55	51	44	35	34	33	31
NPV	69	70	72	73	78	80	84	92	92	92	90	100
Sens	2	15	20	41	49	66	88	90	95	95	100	100
Spec	100	100	99	97	92	88	76	62	48	24	19	11

Data presented as %

A cut off of 2 divided the current, derivation cohort into < 20% or ≥ 20% mortality

Using a score > 2 on the historical, validation dataset, 161 died out of 393 at risk, compared with an expected number of 223, whilst in those with scores ≤2, 41 died out of 349 at risk, compared with an expected number of 39

In those with a score of 0 in the historical, validation dataset, one death was predicted and observed. A score of 0 had a NPV of 100% with a predicted and observed mortality < 5% though this only incorporated 7.5% (10/133) of the current, derivation cohort and 4.3% of the historical (32/741) cohort

### Validation of mortality risk predictive score

Testing on an independent historical dataset [6] of 741 cases of candidaemia gave an AUC of 0.74. The historical validation data set comprised 28% (216/741) patients in ICU and 48% (356/741) had *C. albicans* candidaemia. The data retained an accurate split in predicted mortality at a score of 2; mortality in patients with a score greater than 2 had a 30-day all-cause mortality of at least 25%. A score of 0 predicted a group with < 5% mortality; there was 1 death amongst 32 patients (3.1%) in the historical dataset (Table 3).

## Discussion

Overall mortality in patients with candidaemia has not improved in the past 20 years despite the introduction of echinocandins, either in Australia, or internationally [5, 6, 22]. In the present study, we present the first data on a risk stratification tool for all-cause mortality in candidaemia. Notably, 41% of patients in the derivation and 29% in the validation cohort were in ICU, suggesting that the model can be generalised to non-critical care settings. A score of > 2; constituting either age > 65 years and at least one score criterion, or ≤ 65 years and more than two criteria from the score, identified a group with high mortality of at least 20% where an aggressive approach is warranted including source control and management of predisposing factors. Additionally, these patients may constitute a high-risk group for inclusion into clinical trials. Conversely a score of 0 was associated with a predicted and observed mortality of < 5%, though this applied to few patients.

The source of candidaemia was gastrointestinal or urologic in a majority (55%) of cases in contrast with previous reports from our group and others, where CVAD-related candidaemia predominated [6, 23]. The urinary tract may be an increasing source of candidaemia [24–26]. Urological interventions such as those aimed to remove stones or stents may induce candidaemia, as observed in our cohort. Since the last report where CVAD-related candidaemia was more common [6], national infection prevention programs have been implemented that target optimisation of hand hygiene and reduction of central line infections via the use of central line insertion bundles [17, 27, 28]. Although time to removal of CVADs did not influence outcome in our cohort, this differs from findings of a previous study where earlier removal of lines correlated with decreased mortality and likely reflects prompt removal upon diagnosis in the current study [10]. Whilst the management of CVADs has improved, the increase in candidaemia from a gastrointestinal source has been reported widely [29, 30]. The finding that absence of recent surgery was associated with mortality may indicate that sources not amenable to surgical intervention did more poorly; data examining surgical intervention would help clarify this in future studies. Furthermore, a substantial proportion of cases from an “unknown source” is likely to originate from the gastrointestinal tract due to the phenomenon of bacterial translocation associated with critical illness [31]. A gastro-intestinal or unknown source was an important predictor of mortality, confirming that source is pivotal to outcome [9, 12].

Previous data have linked source with *Candida* species [29, 30, 32] and in the present series the *C. glabrata* complex was associated with a gastrointestinal source; although not advanced age or co-morbidities, as has been reported by others [26, 29, 30]. In Australia, the proportion of candidaemia due to *C. albicans* has fallen further (39%; compared to 47% in 2001–2004 [6]) regardless of setting. Non-*albicans Candida* spp. predominated, reflecting a global trend [4, 5].

Complicated candidaemia (the presence of metastatic infection) was reported in 10% of episodes and rates of endocarditis and endophthalmitis were consistent with those reported previously [6, 15, 23, 33]. As not all patients underwent echocardiography and ophthalmological assessment these are likely to be underestimated.

Mortality in patients treated with fluconazole versus an echinocandin as initial therapy was not significantly different. This may be particular to settings such as in Australia, with a low prevalence of azole resistance [1] and close attention to source control. Notably, all 10 deaths in 54 patients treated with fluconazole as initial therapy, occurred in patients with a mortality risk predictive score of ≥2.5; this could not be attributed to therapy (standard dosing of fluconazole e.g. 400 mg daily) or the isolates (8 of the respective isolates were susceptible to fluconazole with an MIC< 1 mg/L (data not shown). A mortality prediction tool may highlight patients with a higher risk of death not otherwise recognised.

Sepsis [17] was present in the majority of patients and thus did not discriminate those at higher risk of death. The recently published sepsis definitions could be explored in future studies, however they are yet to come into mainstream use either locally or internationally, and perform less well outside of the ICU setting [34, 35]. The APACHE II score is complex, not routinely calculated for non-ICU patients, and as we aimed to develop a score that could be applied to “all-comers” with candidemia, was not included in the model. Additionally, in the subset of patients in ICU, APACHE II score did not perform as well as our derived mortality risk predictive score (data not shown). Location in ICU at the time of diagnosis independently predicted 30-day mortality and was incorporated into the score.

The mortality risk predictive score was derived from variables known at the time of candidaemia diagnosis. Despite differences between the data in this study and the historical cohort data used for validation of the mortality risk predictive score including a longer time to removal of CVAD and predominance of azole therapy [6], the score retained utility. A score of 0 predicted mortality < 5% though this applied only to 7.5% of the current cohort and 4.3% of the historical cohort. The dichotomous prediction model identified a cohort with < 20% mortality that incorporated 36.6% of the current cohort and 47% of the historical cohort.

Strengths of our study include the multicentre prospective data collection of all sequential adult patients with candidaemia, and validation of the derived mortality risk predictive score on an independent dataset of 741 patients. Limitations include restriction of data collection to large hospitals, limited sample size and validation on a historical dataset. Whilst our data identified prolonged antibiotic use as important in mortality, data regarding bacterial infection were not collected and further analyses including this would be useful. Beta-D glucan was unavailable and procalcitonin was not routinely used in the settings assessed; incorporation of these in future studies may be informative. Future prospective studies would also benefit from inclusion of more centres.

## Conclusions

In conclusion, mortality in patients with candidaemia remains high and we present a novel, simple prediction model that may be applied at a clinically useful time point to stratify predicted mortality of patients with candidaemia. In the present study, non-*albicans Candida* spp. predominated and a gastrointestinal and urologic source of candidaemia were common, demonstrating contemporary changes in the demographics of patients with candidemia.

## Additional file

Additional file 1: Definitions used in the present study and their explanations. Definitions used in study [36]. (DOCX 18 kb)

## Abbreviations

APACHE: Acute physiology and chronic health evaluation
CA: Community acquired
CVAD: Central venous access device
GVHD: Graft versus host disease
ICU: Intensive care unit
IHCA: Inpatient healthcare associated
IQR: Interquartile range
ITS: Internal transcribed spacer
MALDI-TOF MS: Matrix-assisted laser desorption/ionization-time of flight mass spectroscopy
OHCA: Outpatient healthcare associated
TPN: Total parenteral nutrition
