# Supplementary material for: Candidaemia and a risk predictive model for overall mortality: a prospective multicentre study
Source: BMC Infect Dis. 2019 May 21;19:445. doi: 10.1186/s12879-019-4065-5 (PMC6528341; doi:10.1186/s12879-019-4065-5)
Supplement: Supplementary file 1 — Definitions used in the present study and their explanations. Definitions used in study [36]. (DOCX 18 kb) [file 12879_2019_4065_MOESM1_ESM.docx]

# Appendix

### Definitions used in the present study and their explanations

**1. Setting** (6, 36)

Inpatient healthcare associated (IHCA) candidaemia: episodes occurring >48hours after hospital and not clinically manifest on admission

Outpatient healthcare associated (OHCA) candidaemia: episodes manifest on admission or within 48hours but associated with a healthcare associated event e.g. antecedent surgery, placement of an indwelling medical device or neutropaenia (<1.0x10^9^/L) after cytotoxic chemotherapy

Community acquired (CA) candidaemia: episodes manifest on admission or within 48hours and with no healthcare-related predisposing factors

**2. Source of candidemia:**

| Assigned source | Criteria |
| --- | --- |
| Intravascular | Same *Candida* species isolated from the tip of an intravascular device as that from blood culture  OR  Physician-ascribed where indwelling intravascular device was the only likely source  OR  Candidaemia in the context of documented intravenous drug use as the only likely source |
| Gastrointestinal | Same *Candida* species identified from specimens originating from a gastrointestinal or intra-abdominal source as that from blood culture  OR  Physician-ascribed where documented interventional procedure e.g. gastrointestinal surgery, biliary tract manipulation and no other likely source |
| Urologic | Same *Candida* species isolated from urine as that from blood culture following antecedent urologic instrumentation  OR  Surgery that clearly preceded candidaemia and no other likely source |
| Unknown | Not attributable to an intravascular, gastrointestinal or urologic source |

**3. Patient comorbidity**: These refer to underlying medical conditions present at the time of candidaemia

| Medical condition | Definition |
| --- | --- |
| Acute leukaemia | Acute lymphoproliferative malignancy with >20% blasts in the peripheral blood or bone marrow |
| Allogeneic SCT | Allogeneic stem cell transplant |
| Autologous SCT | Autologous stem cell transplant |
| Cardiovascular disease | Severe congestive heart failure. Symptoms at rest/inability to carry on physical activity without discomfort |
| Chronic leukaemia | Chronic lymphoproliferative malignancy with >20% blasts in the peripheral blood or bone marrow |
| Chronic organ dysfunction | Renal disease, liver disease, cardiovascular disease or respiratory disease |
| Connective tissue | Autoimmune condition including systemic lupus erythematosus, rheumatoid arthritis, vasculitis |
| Diabetes mellitus | Elevated HbA1c >6.5% |
| Haematologic malignancy | Any haematologic malignancy |
| Haemotology cancer other | Haematological cancer other than acute leukaemia, chronic leukaemia, lymphoma |
| HIV/AIDS | HIV confirmed by western blot, p24 antigen and/or HIV proviral DNA |
| HSCT | Autologous or allogeneic SCT |
| Liver disease | Biopsy proven cirrhosis with portal hypertension and/or past upper GIT bleeding due to portal hypertension and/or prior episodes of hepatic failure/encephalopathy/coma |
| Lymphoma | Clonal lymphocytes |
| Renal disease | On chronic haemodialysis or peritoneal dialysis |
| Respiratory disease | Disease severely restricting exercise e.g. inability to climb stairs or do household duties and/or documented chronic respiratory hypoxia and/or pulmonary hypertension |
| Solid organ cancer | Active solid organ malignancy |
| Solid organ transplantation | Renal, cardiac, lung, liver or pancreatic organ transplantation |

**4. Predisposing factors**: These refer to predisposing factors present at or within the last 30 days prior to candidemia. Factors sought for:

Any major surgery (eg. hepatobiliary, gastrointestinal, urological produres), Central venous access device (CVAD), indwelling urinary catheter or other urinary drainage device, intravenous drug use, receipt of total parenteral nutrition, haemodialysis, receipt of corticosteroids, other immunosuppressive therapy, cytotoxic chemotherapy, major trauma, receipt of broad spectrum antibiotic agents: any antibiotic agent prescribed for more than 2 days before onset of candiaemia

**5. Prior antifungal use**: Prophylactic antifungal therapy administered before onset of candidaemia; excludes use of antifungal therapy for treatment of candidaemia
